# A Locust-Inspired Model of Collective Marching on Rings

**DOI:** 10.3390/e24070918

**Published:** 2022-06-30

**Authors:** Michael Amir, Noa Agmon, Alfred M. Bruckstein

**Affiliations:** 1 Department of Computer Science, Technion—Israel Institute of Technology, Haifa 3200003, Israel; freddy@technion.ac.il; 2 Department of Computer Science, Bar-Ilan University, Ramat Gan 5290002, Israel; agmon@cs.biu.ac.il

**Keywords:** mobile robotics, swarms, crowd dynamics, natural algorithms, locusts

## Abstract

We study the collective motion of autonomous mobile agents in a ringlike environment. The agents’ dynamics are inspired by known laboratory experiments on the dynamics of locust swarms. In these experiments, locusts placed at arbitrary locations and initial orientations on a ring-shaped arena are observed to eventually all march in the same direction. In this work we ask whether, and how fast, a similar phenomenon occurs in a stochastic swarm of simple locust-inspired agents. The agents are randomly initiated as marching either clockwise or counterclockwise on a discretized, wide ring-shaped region, which we subdivide into *k* concentric tracks of length *n*. Collisions cause agents to change their direction of motion. To avoid this, agents may decide to switch tracks to merge with platoons of agents marching in their direction. We prove that such agents must eventually converge to a local consensus about their direction of motion, meaning that all agents on each narrow track must eventually march in the same direction. We give asymptotic bounds for the expected time it takes for such convergence or “stabilization” to occur, which depends on the number of agents, the length of the tracks, and the number of tracks. We show that when agents also have a small probability of “erratic”, random track-jumping behavior, a global consensus on the direction of motion across all tracks will eventually be reached. Finally, we verify our theoretical findings in numerical simulations.

## 1. Introduction

Birds, locusts, human crowds, and swarm-robotic systems exhibit interesting collective motion patterns. The underlying autonomous agent behaviors from which these patterns emerge have attracted a great deal of academic interest over the last several decades [1,2,3,4,5,6]. In particular, the formal analysis of models of swarm dynamics has led to varied and deep mathematical results [7,8,9,10]. Rigorous mathematical results are necessary for understanding swarms and for designing predictable and provably effective swarm-robotic systems. However, multi-agent swarms have a uniquely complex and “mesoscopic” nature [11], and relatively few standard techniques for the analysis of such systems have been established. Consequently, the analysis of new models of swarm dynamics is important for advancing our understanding of the subject.

**Figure 1:**
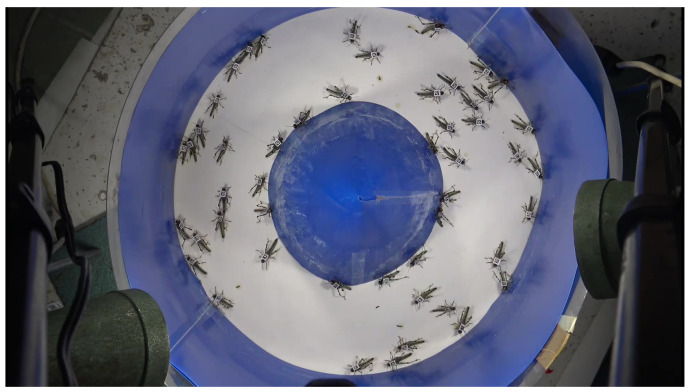
Image from locust experiments, courtesy of Amir Ayali. The collective clockwise marching of locusts in a ring arena is shown. Locusts were initiated at random positions and orientations in the arena but converged to clockwise marching over time.

In this work, we study the dynamics of “locust-like” agents moving on a discrete ringlike surface. The model we study is inspired by the following well-documented experiment [12]. Place many locusts on a ringlike arena at random positions and orientations. They start to move around and bump into the arena’s walls and into each other, and as they do, remarkably, over time, they begin to collectively march in the same direction–either clockwise or counterclockwise (see Figure 1). Inspired by observing these experiments, we asked the following question: What are simple and reasonable myopic rules of behavior that might lead to this phenomenon? Our goal is to study this question from an *algorithmic* perspective by considering a swarm of autonomous and identical discretized mobile agents that act according to a local algorithm. The precise mechanisms underlying locusts’ behaviors are very complex and subject to intense ongoing research, e.g., [3,12,13,14,15]. Consequently, as with much of the literature on swarm dynamics [7,16,17], our goal is not to study an exact mathematical model of locusts in particular, but to study the kinds of algorithmic local interactions that lead to collective marching and related phenomena. The resulting model is idealized and simple to describe, but the patterns of motion that emerge while the locusts progress toward a “stabilized” state of collective marching are surprisingly complex.

The starting point for this work is the following postulated “rationalization” of what a locust-like agent wants to do. It wants to keep moving in the same direction of motion (clockwise or counterclockwise) for as long as possible. We can therefore consider a model of locust-like agents that never change their heading unless they collide head-on with agents marching in the opposite direction and are forced to do so due to the pressure which is exerted on them. When possible, these agents prefer to bypass agents that are headed toward them rather than collide with those agents. This is accomplished by changing lanes, moving in an orthogonal manner between concentric narrow *tracks* which partition the ringlike arena. The formal description of this “rationalized” model is given in Section 3, and will be our subject of study.

### Contribution

We describe and study a stochastic model of locust-inspired agents in a 2D discretized ringlike arena which is subdivided into *k* tracks, each consisting of *n* locations. We show that our agents eventually reach a “local consensus” about the direction of marching, meaning that all agents on the same track will march in the same direction. We give asymptotic bounds for the time this takes based on the number of agents and the physical dimensions of the arena. Due to the idealized deterministic nature of our model, a global consensus where *all* locusts walk in the same direction is not guaranteed, since locusts in different tracks might never meet. However, we show that when a small probability of “erratic”, random behavior is added to the model, such a global consensus must occur. We verify our claims via simulations and make further empirical observations that may inspire future investigations into the model.

Despite being simple to describe, analyzing the model proved tricky in several respects. Our analysis strategy is to show that the model oscillates between two phases: one in which it is “chaotic”, and locusts are moving about without a discernible pattern; and one in which it is “orderly”, and all locusts are stuck in dense deadlock situations where collisions are frequent. We derive our asymptotic bounds from studying orderly phases while bounding the amount of time the locusts can spend in the chaotic phases.

Previous works in the literature (e.g., [18,19]) have explained collective marching by appealing to a principle of local averaging, wherein each agent attempts to average its direction of motion with its neighbors’. It is interesting to note that our model attains collective marching from nearly the opposite set of assumptions. Our agents’ primary motivation is to *avoid* changing their direction of motion, and any change to it is thus the result of an unavoidable conflict. We refer the reader to the Related Work section below for further discussion.

## 2. Related Work

Some of our results were presented at DARS2021 [20]. Here we extend our work by improving the time bounds for reaching consensus (Theorem 2) by substantially expanding and restructuring the technical analysis to include the full details which were omitted in [20] and by adding new simulation results, technical figures, and references.

The locust experiments inspiring our work are discussed in [3,12,13,14,18,19]. The phenomenon was originally studied by Buhl et al. [18]. They show that above a certain critical density, a rapid transition occurs from disordered movements of locust nymphs to highly aligned collective motion. Buhl et al., and subsequently Yates et al. [19], hypothesize that a main cause of this behavior is the locusts’ tendency to change their direction to align with neighbors within a local interaction range (a common modeling assumption in multi-agent dynamics [9]) and that individual behavior does not change in relation to group density. In this work, we show how collective marching might emerge from almost the opposite set of assumptions; the locust-like agents we describe try to *avoid* changing their direction of motion for as long as possible, going as far as actively avoiding locusts that are headed in the opposite direction However, consensus eventually occurs as a result of unavoidable conflicts where locusts bump into each other. The assumption that locusts want to maintain their direction of motion is critical for enabling collective marching in our model, since it characterizes the stable states of the system. Bazazi et al. [21] hypothesize collective marching occurs due to a model of cannibalistic pursuit wherein locusts attempt to pursue locusts in front of them and evade locusts behind them to bite and avoid being bitten. Our model includes an element of evasion, too, but it is motivated by the locusts’ desire to avoid changing their direction of motion. All previous models assume local interactions between locusts, i.e., locusts are only affected by neighboring locusts. Interactions in our model consist of conflicts between adjacent locusts and track changes that occur as a result of trying to avoid said conflicts. Conflicts are by definition local. Track-changing rules can be assumed either local or global, and our analysis applies in both cases.

Notably, in [18,19], it is observed that at intermediate densities swarms of locusts exhibit periodic directional switching, and at low densities the directions of motion are random. Our model does not replicate these phenomena; we show that our locust-like agents converge to local consensus at every density (or global consensus, assuming noise). Interestingly, we note that if we assume each locust has a small probability r>0 of randomly flipping their heading at the beginning of a time step, such directional switching becomes possible. The probability of directional switching under such a postulate is inversely proportional to the density, thus likelier at low and intermediate densities than at high densities. We emphasize, however, that unlike works such as Buhl et al., replicating all features of locust swarms is not the goal of this work. Whereas the works we discussed seek to model actual locusts, our work can be characterized as trying to find a minimalistic locust-*inspired* set of assumptions that provably attains collective marching and to study it analytically for the sake of deepening our understanding of multi-agent systems.

More generally, the mathematical modeling of the collective motion of natural organisms, such as birds, locusts, and ants, and the convergence of such systems of agents to stable formations, has been discussed in numerous works including [5,9,10,22]. The most relevant to us among these are works within the field of *natural algorithms*, which assert that the behavior of natural organisms can be understood using concepts from the theory of robotics and computer science [11,16,17], such as complexity analysis, look-compute-move phases, and decision-making based on discrete internal states. Natural algorithms open up interplay between biology and computer science, allowing us to study nature via the language of algorithms and vice versa, allowing us to translate principles, algorithms, and mechanisms gleaned from nature to the design of systems that are meant to service or interact with humans, such as autonomous vehicles and warehouse robots.

The central focus of this work regards consensus. Do the agents eventually converge to the same direction of motion, and how long does it take? These questions bear mathematical and conceptual resemblance to questions in the field of opinion dynamics [23,24,25]. If the agents’ direction of motion (clockwise or counterclockwise) is considered an “opinion”, and the agents’ interactions that cause changes in the direction of motion are considered social pressure, we can ask how long it takes for the agents to arrive at a consensus of opinions. Building on this analogy, we note that when there are no empty locations in the environment, our agent model is distinctly similar to the *voter model* on a ring network with two opinions. The voter model is a classical model in opinion dynamics explored in numerous works (we refer the reader to the survey [26]).

The comparison to the voter model breaks when we introduce empty locations and multiple ringlike tracks at which point we must take into account the agents’ dynamically changing positions. Unlike the voter model, where only an agent’s static neighborhood can influence its opinion, in our model, an agent’s current location determines which agents can influence it. Several works have explored models of opinion dynamics in a ring environment where the agents’ physical location is taken into account [27,28]. Our model is distinct from these in several respects. First, in our model, an agent’s internal state—its direction of motion—plays an active part in the algorithm that determines which locations an agent may move to. Second, we partition our ring topology into several narrow rings (“tracks”) that agents may switch between, and an agent’s decision to switch tracks is influenced by the presence of platoons of agents moving in its direction in the track that it wants to switch to. In other words, we model agents that actively attempt to “swarm” together with agents moving in their direction of motion. We believe our work is unique in that we study, in a single model, both how an agent’s physical location affects its opinion (via conflicts with nearby agents), and how an agent’s opinion affects its physical location (via the desire to swarm with agents of the same opinion or equivalently, evade those of a different opinion).

Protocols for achieving consensus about a value, location, or the collective direction of motion have also been investigated in swarm robotics and distributed algorithms [29,30,31,32]. The purpose of these protocols is typically to be as efficient as possible in terms of parameters such as time, computational load, and distance traveled. However, in this work, we are not searching for a protocol that is designed to efficiently bring about consensus; we are investigating a protocol that is inspired by natural phenomena and want to see *whether* it leads to consensus and how long this process is expected to take.

Broadly speaking, some mathematical similarities may be drawn between our model and interacting particle systems such as the simple exclusion process, which has been used to understand biological transport and traffic phenomena [33,34]. Such particle systems have been studied on rings [35]. In these discrete models, as in our model, agents possess a physical dimension, which constrains the locations they might move to in their environment. These are not typically multi-agent models where agents have an internal state (such as a persistent direction of motion), but rather models of particle motion and diffusion, and the research focus is quite different. The main point of similarity to our model is in the way that a given discrete location can only be occupied by a single agent and in the random occurrence of “traffic shocks” where agents line up one after the other and are prevented from moving for a long time.

## 3. Model and Definitions

**Figure 2:**
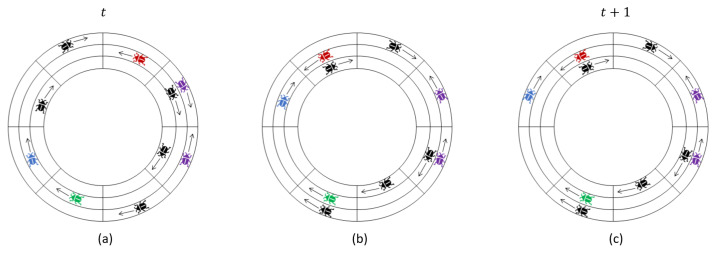
One step of the locust model with k=3, n=8 split into horizontal and vertical movements: (**a**) shows the initial configuration at the beginning of the current time step *t*; (**b**) illustrates changes to the configuration after conflicts and horizontal movements; and (**c**) is the configuration at the beginning of time t+1 (or equivalently the end of time *t*) after vertical movements. The *front* and *back* of the blue locust are the red and green locusts, respectively. The purple locusts conflict with each other. Since conditions (1)–(3) are fulfilled, the blue locust may switch tracks, and it does so in the illustration.

We postulate a locust-inspired model of marching in a wide 2D ringlike arena which is discretized into *k* narrow concentric rings, each consisting of *n* locations. Each narrow concentric ring is called a *track*. This discretized environment is *topologically* equivalent to the surface of a discretized cylinder of height *k* partitioned into *k* narrow rings of length *n* which are layered on top of each other. For example, the environment of Figure 2 corresponds to k=3,n=8 (3 tracks of length 8). The coordinate (x,y) refers to the *x*th location on the *y*th track (which can also be seen as the *x*th location of a ring of length *n* wrapped around the cylinder at height *y*). We define ∀x,(x+n,y)≡(x,y).

A swarm of *m* identical agents, or “locusts", which we label $A_{1},\ldots ,A_{m}$, are dispersed at arbitrary locations and move autonomously at discrete time steps t=0,1,…. A given location (x,y) can contain at most one locust. Each locust $A_{i}$ is initiated with either a “clockwise” or “counterclockwise” *heading*, which determines their present direction of motion. We define $b(A_{i})=1$ when $A_{i}$ has clockwise heading, and $b(A_{i})=-1$ when $A_{i}$ has a counterclockwise heading.

The locusts move synchronously at discrete time steps t=0,1,…. At every time step, locusts try to take a step in their direction of motion. If locust *A* is at (x,y), it will attempt to move to (x+b(A),y). A clockwise movement corresponds to adding 1 to *x*, and a counterclockwise movement corresponds to subtracting 1. The locusts have physical dimension, so if the location a locust attempts to move to already contains another locust at the beginning of the time step, the locust instead stays put. If $A_{i}$ and $A_{j}$ are both attempting to move to the same location, one of them is chosen uniformly at random to move to the location, and the other stays put.

Locusts that are adjacent exert pressure on each other to change their heading. If $A_{i}$ has a clockwise heading and $A_{j}$ has a counterclockwise heading, and they lie on the coordinates (x,y) and (x+1,y), respectively, then at the end of the current time step, one locust (chosen uniformly at random) will flip its heading to the other locust’s heading. An equivalent way to model these dynamics is as follows: at the start of a conflict, each of the two locusts uniformly samples a random number $r_{i},r_{j}\in \left(0,1\right)$ called ’pressure’. The locust with lower pressure “loses” the conflict and changes its heading (noting that the probability of $r_{i}=r_{j}$ is 0). Such an event is called a **conflict** between $A_{i}$ and $A_{j}$. A conflict is “won” by the locust that successfully converts the other locust to their heading.

Let *A* be a locust at (x,y). If the locust *A* has a clockwise heading, then the *front* of *A* is the first locust after *A* in the clockwise direction, and the *back* of *A* is the first locust in the counterclockwise direction. The reverse is true when *A* has a counterclockwise heading. Formally, let i>0 be the smallest positive integer such that (x+b(A)i,y) contains a locust, and let j>0 be the smallest positive integer such that (x−b(A)j,y) contains a locust. The *front* of *A* is the locust in (x+b(A)i,y), and the *back* of *A* is the locust in (x−b(A)j,y). The locusts in the front and back of *A* are denoted $A^{\to }$ and $A^{\leftarrow }$, respectively, and are called *A*’s *neighbors*; these are the locusts that are directly in front of and behind *A*. Note that when a track has two or less locusts, $A^{\to }=A^{\leftarrow }$. When a track has one locust, i=j=n, and $A=A^{\to }=A^{\leftarrow }$.

At any given time step, besides moving in the direction of their heading within their track, locust *A* at (x,y) can switch tracks, moving vertically from (x,y) to (x,y+1) or (x,y−1) unless this would cause it to go above track *k* or below track 1. Such vertical movements occur *after* the horizontal movements of locusts along the tracks but on the same time step where those horizontal movements took place. Locusts are incentivized to move vertically when this enables them to avoid changing their heading (“inertia”). Specifically, *A* may move to the location E=(x,y±1) at time *t* when:

1. At the beginning of time *t*, *A* and $A^{\to }$ are not adjacent to each other and $b\left(A\right)\neq b\left(A^{\to }\right)$.
2. Once *A* moves to *E*, the updated $A^{\leftarrow }$ and $A^{\to }$ in the new track will have heading b(A).
3. No locust will attempt to move horizontally to *E* at time t+1.

Condition (1) states that there is an imminent conflict between *A* and $A^{\to }$ which is bound to occur. Condition (2) guarantees that, by changing tracks to avoid this conflict, *A* is not immediately advancing toward another collision; *A*’s new neighbor will have the same heading as *A*. Condition (3) guarantees that the location *A* wants to move to on the new track is not being contested by another locust already on that track. Together, these conditions mean that locusts only change tracks if this results in avoiding collisions and in “swarming” together with other locusts marching in the same direction of motion. If a locust cannot sense that all three conditions (1)–(3) are fulfilled, it does not switch tracks.

Besides these conditions, we make no assumptions about *when* locusts move vertically. In other words, locusts do not always need to change tracks when they are allowed to by rules (1)–(3); they may do so arbitrarily, say with some probability *q* or according to any internal scheduler or algorithm, and we may impose visibility range constraints on the locusts such that they only switch tracks when they can *see* that rules (1)–(3) are fulfilled. We do not determine in any sense the times when locusts move between tracks, but only determine the preconditions required for such movements. Our results in the following sections remain true regardless. This makes our results general in the sense that they hold for many different track-switching “swarming” rules, as long as those rules do not break the conditions (1)–(3).

Figure 2 illustrates one time step of the model, split into horizontal and vertical movement phases.

To slightly simplify our analysis of the model, we assume that every track has at least two locusts at all times, although our results remain true without this assumption.

Although we work in a discrete time model where movement is instantaneous, it is helpful for the sake of formal analysis to define *the beginning* of a time step as the configuration of the swarm at that time step before any locusts moved, and *the end* of a time step as the configuration at that time step after all locust movements are complete. Somewhat idiosyncratically, the end of time *t* is precisely the beginning of time t+1—both terms refer to the same thing. By default and unless stated otherwise, the words “time step *t*” refer to the beginning of that time step.

## 4. Stabilization Analysis

We will mainly be interested in studying the stability of the headings of the locusts over time. Does the model reach a point where the locusts stabilize and stop changing their heading? If so, are their headings all identical? How long does it take?

In the case of a single track (k=1), we shall see that the locusts all eventually stabilize with identical heading and bound the expected time for this to happen in terms of *m* and *n*. In the multi-track case, we shall see that the locusts stabilize and agree on a heading *locally* (i.e., all locusts *on the same track* eventually have the identical heading and thereafter never change their heading) and bound the expected time to stabilization in terms of m,n,k. In the multi-track case, we further show that adding a small probability of “erratic” track-switching behavior to the model induces *global* consensus: all locusts across all tracks eventually have the identical heading.

### 4.1. Locusts on Narrow Ringlike Arenas (k=1)

We start by studying the case k=1; that is, we study a swarm of *m* locusts marching on a single track of length *n*. Throughout this section, we assume this is the case, except in Definition 1, which is also used in later sections.

For the rest of this section, let us call the swarm *non-stable* at time *t* if there are two locusts $A_{i}$ and $A_{j}$ such that $b\left(A_{i}\right)\neq b\left(A_{j}\right)$; otherwise, the swarm is *stable*. A swarm which is stable at time *t* remains stable thereafter. We wish to bound the number of time steps it takes for the system to become stable, which we denote $T_{stable}$. Our goal is to prove Theorem 1, which tells us that the expected time to stabilization grows quadratically in the number of locusts *m* and linearly in the track length *n*.

**Theorem** **1.** *For any configuration of m locusts on a ring with a single track, $E\left[T_{stable}\right]\leq m^{2}+2\left(n-m\right)$. This bound is asymptotically tight; there are initial locust configurations for which $E\left[T_{stable}\right]=\Omega \left(m^{2}+n-m\right)$.*

Theorem 1 tells us that all locusts must have identical bias within a finite expected time. This fact in isolation (without the time bounds in the statement of the theorem) is relatively straightforward to prove by noting that the evolution of the locusts’ headings and locations can be modeled as a finite Markov chain, and the only absorbing classes in this Markov chain are ones in which all locusts have the same heading (see [36]).

**Figure 3:**
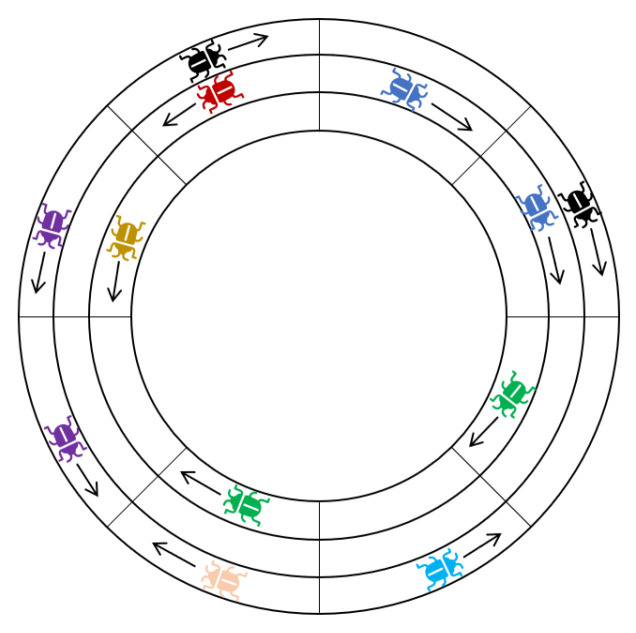
A locust configuration with n=8,k=3. Locusts are colored based based on the segment they belong to (Definition 1). There are 8 segments in total.

Next we define *segments*: sets of consecutive locusts on the same track which all have the same heading. This allows us to partition the swarm into segments such that every locust belongs to a unique segment (see Figure 3). Although this section focuses on the case of a single track (and claims in this section are made under the assumption that there is only a single track), the definition is general, and we will use it in subsequent sections.

**Definition** **1.** *Let A be a locust for which $b\left(A^{\leftarrow }\right)\neq b\left(A\right)$ at time t, and consider the sequence of locusts $B_{0}=A$, $B_{i+1}=B_{i}^{\to }$. Let $B_{q}$ be the first locust in this sequence for which $b\left(B_{q}\right)\neq b\left(B_{0}\right)$. The set $\left\{B_{0},B_{1},\ldots B_{q-1}\right\}$ is called the* ***segment** of the locusts $B_{0},\ldots B_{q-1}$ at time t. The locust $B_{q-1}$ is called the* ***segment head**, and A is called the* ***segment tail** of this segment.*

Only locusts which are segment heads at the beginning of a time step can change their heading by the end of that time step. When the heads of two segments are adjacent to each other, the resulting conflict causes one to change its heading, leave its previous segment, and instead become part of the other segment. If the head of a segment is also the tail of a segment, the segment is eliminated when it changes heading. Two segments separated by a segment of opposite heading merge if the opposite-heading segment is eliminated, which decreases the number of segments by two. No other action by a locust can change the segments. Hence, the number of segments and segment tails can only decrease.

Since our model is stochastic, different sequences of events may occur and result in different segments. However, by the above argument, we can conclude that in any such sequence of events, there must always exist at least one locust which remains a segment tail at all times $t<T_{stable}$ and never changes its heading (since at least one segment must exist as long as $t<T_{stable}$). Arbitrarily, we denote one such segment tail “$A_{W}$”.

**Definition** **2.** *The segment of $A_{W}$ at the beginning of time t is called the* ***winning segment** at time t and is denoted SW(t). The head of SW(t) is labelled $H_{W}\left(t\right)$. For convenience, if at time $t_{0}$ the swarm is stable (i.e., $t_{0}\geq T_{stable}$), then we define $SW(t_{0})$ as the set that contains all m locusts.*

**Lemma** **1.** *The expected number of time steps $t<T_{stable}$ in which |SW(t)| changes is bounded by $m^{2}$.*

**Proof.**  Let $C_{m}$ denote the number of changes to the size of SW(t) that occurs before time $T_{stable}$. Note that $T_{stable}$ is the first time step where |SW(t)|=m. |SW(t)| can only decrease, by one locust at a time, if $H_{W}\left(t\right)$ conflicts with another locust and loses. |SW(t)| can increase in several ways, for example, when it merges with other segments. In particular, |SW(t)| increases by at least one whenever $H_{W}\left(t\right)$ conflicts with a locust and wins, which happens with probability at least $\frac{1}{2}$. Hence, whenever SW(t) changes in size, it is more likely to grow than to shrink. We can bound $E[C_{m}]$ by comparing the growth of |SW(t)| to a random walk with absorbing boundaries at 0 and *m*:

Consider a random walk on the integers which starts at |SW(0)|. At any time step *t*, the walker takes a step left with probability $\frac{1}{2}$, otherwise it takes a step right. If the walker reaches either 0 or *m*, the walk ends. Denote by $C_{m}^{*}$ the time it takes the walk to end. Using *coupling* (cf. [37]), we see that $E\left[C_{m}\right]\leq E\left[C_{m}^{*}|\mathrm{the}\;\mathrm{walker}\;\mathrm{never}\;\mathrm{reaches}\;0\right]$, since per the previous paragraph, |SW(t)| clearly grows at least as fast as the position of the random walker (note that |SW(t)|>0 is always true, which is analogous to the walker never reaching 0).

Let us show how to bound $E[C_{m}^{*}|\mathrm{the}\;\mathrm{walker}\;\mathrm{never}\;\mathrm{reaches}\;0]$. Since the walk is memoryless, we can think of this quantity as the number of steps the random walker takes to get to *m*, assuming it must move right when it is at 0, and assuming the step count restarts whenever it moves from 0 to 1. If we count the steps without resetting the count, we realize that this is simply the expected number of steps it takes a random walker walled at 0 to reach position *m*, which is at most $m^{2}$ (cf. [38]). Hence $E\left[C_{m}^{*}|\mathrm{the}\;\mathrm{walker}\;\mathrm{never}\;\mathrm{reaches}\;0\right]\leq m^{2}$. □

**Lemma** **2.** *The expected number of time steps $t<T_{stable}$ in which |SW(t)| does not change is bounded by 2(n−m).*

Lemma 2 will require other lemmas and some new definitions to prove.

**Definition** **3.** *Let A and B be two locusts or two locations which lie on the same track. The clockwise distance from A to B at time t is the number of clockwise steps required to get from A’s location to B’s location and is denoted $dist^{c}\left(A,B\right)$. The counterclockwise distance from A to B is denoted $dist^{cc}\left(A,B\right)$ and equals $dist^{c}\left(B,A\right)$.*

For the rest of this section, let us assume without loss of generality that the winning segment’s tail $A_{W}$ has a clockwise heading. Label the empty locations in the ring at time t=0 (i.e., the locations not containing locusts at time t=0) as $E_{1},E_{2},\ldots E_{n-m}$, sorted by their counterclockwise distance to $A_{W}$ at time t=0, such that $E_{1}$ minimizes $dist^{cc}\left(E_{i},A_{W}\right)$, $E_{2}$ has the second smallest distance, and so on. We will treat these empty locations as having persistent identities. Whenever a locust *A* moves from its current location to $E_{i}$, we will instead say that *A* and $E_{i}$*swapped*, and so $E_{i}$’s new location is *A*’s old location.

We say a location $E_{i}$ is *inside* the segment SW(t) at time *t* if the two locusts which have the smallest clockwise and counterclockwise distance to $E_{i}$, respectively, are both in SW(t). Otherwise, we say that $E_{i}$ is *outside* SW(t). A locust or location *A* is said to be *between* $E_{i}$ and $E_{j}$, j>i, if $dist^{c}\left(E_{i},A\right)<dist^{c}\left(E_{i},E_{j}\right)$.

**Definition** **4.** *All empty locations are initially* ***blocked**. A location $E_{i}$ becomes* ***unblocked** at time t+1 if all empty locations $E_{j}$ such that j<i are unblocked at time t, and a locust from SW(t) swapped locations with $E_{i}$ at time t. Once a location becomes* ***unblocked**, it remains that way forever.*

**Lemma** **3.** There is some time step $t^{*}\leq n-m$ such that:

*1.*  Every blocked empty location E is outside $SW(t^{*})$ (if any exist)
*2.*  At least $t^{*}$ empty locations are unblocked.

**Proof.**  If $E_{1}$ is outside SW(0), then the same must be true for all other empty locations, so $t^{*}=0$ and we are finished. Otherwise, $E_{1}$ becomes unblocked at time t=1. If $E_{i}$ becomes unblocked at time *t*, then at time *t*, it cannot be adjacent to $E_{i+1}$, since the locust that swapped with $E_{i}$ in the previous time step is now between $E_{i}$ and $E_{i+1}$. By definition, there are no empty locations $E_{j}$ between $E_{i}$ and $E_{i+1}$. Consequently, if $E_{i+1}$ is inside SW(t) at time *t*, it will swap with a locust of SW(t) at time *t*, and become unblocked at time t+1. If $E_{i+1}$ is outside the segment at time *t*, it will become unblocked at the first time step $t^{\prime }>t$ that begins with $E_{i+1}$ inside $SW(t^{\prime })$. Hence, if $E_{i}$ becomes unblocked at time *t*, then $E_{i+1}$ becomes unblocked at time t+1 or $E_{i+1}$ is outside SW(t+1) at time t+1.

Let $t^{*}$ be the smallest time where there are no blocked empty locations inside $SW(t^{*})$. By the above, at every time step $t\leq t^{*}$ an empty location becomes unblocked; hence there are at least $t^{*}$ unblocked empty locations at time $t^{*}$. Moreover, since there are n−m empty locations, this implies $t^{*}\leq n-m$. □

**Lemma** **4.** *There is no time $t<T_{stable}$ where an unblocked location is clockwise-adjacent to $H_{W}\left(t\right)$ (i.e., there is no time t where an unblocked empty location E is located one step clockwise from $H_{W}\left(t\right)$).*

**Proof.**  First consider what happens when $E_{1}$ becomes unblocked: it swaps its location with a locust in SW(t), and since $E_{1}$ is the clockwise-closest empty location to $A_{W}$, the entire counterclockwise path from $E_{1}$ to $A_{W}$ consists only of locusts from SW(t). Hence $E_{1}$ will move counterclockwise at every time step until it swaps with $A_{W}$. Once it swaps with $A_{W}$, $E_{1}$ will not swap with another locust at all times $t<T_{stable}$, since for that to occur we must have that $b\left(A_{W}^{\leftarrow }\right)=b\left(A_{W}\right)$, which is impossible since by definition $A_{W}$ remains a segment tail until $t=T_{stable}$. $E_{1}$ does not swap with $H_{W}\left(t\right)$ while $E_{1}$ moves counterclockwise toward $A_{W}$ nor after $E_{1}$ and $A_{W}$ swap as long as the swarm is unstable; hence there is no time step $t<T_{stable}$ when $E_{1}$ is unblocked and swaps with $H_{W}\left(t\right)$.

Now consider $E_{2}$. $E_{2}$ becomes unblocked at least one time step after $E_{1}$, and there is at least one locust in SW(t) which is between $E_{1}$ and $E_{2}$ at the time step $E_{1}$ that becomes unblocked (in particular, the locust in SW(t) that swapped with $E_{1}$ must be between $E_{1}$ and $E_{2}$ at that time). Since $E_{1}$ subsequently moves toward $A_{W}$ at every time step until they swap, $E_{2}$ cannot become adjacent to $E_{1}$ until they both swap with $A_{W}$. Hence the location one step counterclockwise to $E_{2}$ must always be a locust until $E_{2}$ swaps with $A_{W}$, meaning that similar to $E_{1}$, $E_{2}$ also moves counterclockwise toward $A_{W}$ at every time step after $E_{2}$ becomes unblocked until they swap locations. Consequently, just like $E_{1}$, there is no time step $t<T_{stable}$ when $E_{2}$ is unblocked and swaps with $H_{W}\left(t\right)$.

More generally, by a straightforward inductive argument, the exact same thing is true of $E_{i}$: once it becomes unblocked, it moves counterclockwise toward $A_{W}$ at every time step until it swaps with $A_{W}$. Thus, upon becoming unblocked, $E_{i}$ does not swap with $H_{W}\left(t\right)$ as long as $t<T_{stable}$. □

Using Lemmas 3 and 4, let us prove Lemma 2.

**Proof.**  If, at the beginning of time step *t*, $H_{W}\left(t\right)$ is adjacent to a locust from a different segment, then |SW(t)| will change at the end of this time step due to the locusts’ conflict. Hence, to prove Lemma 2, it suffices to show that out of all the time steps before time $T_{stable}$, $H_{W}\left(t\right)$ is not adjacent to the head of a different segment in at most 2(n−m) different steps in expectation.

If all empty locations are unblocked at time n−m, then by Lemma 4, $H_{W}\left(t\right)$ conflicts with the head of another segment at all times t≥n−m. Therefore, |SW(t)| will change at every time step $n-m<t<T_{stable}$, which is what we wanted to prove.

If there is a blocked location at time n−m, then by Lemma 2, there must be some time $t^{*}\leq n-m$ where at least $t^{*}$ empty locations are unblocked and all blocked empty locations are outside $SW(t^{*})$. Let $E_{j}$ be the minimal-index blocked location which is outside $SW(t^{*})$ at time $t^{*}$. Since there are no blocked empty locations inside $SW(t^{*})$, all locations $E_{i}$ with i<j are unblocked. Hence, $E_{j}$ will become unblocked as soon as it swaps with the head of the winning segment. Since (by the clockwise sorting order of $E_{1},E_{2},\ldots$) $E_{j+1}$ cannot swap with the winning segment head before $E_{j}$ is unblocked, $E_{j+1}$ will also become unblocked after the first time step where it swaps the winning segment head. The same is true for $E_{j+2},\ldots E_{n-m}$. Hence, every empty location that $H_{W}\left(t\right)$ swaps with after time $t^{*}$ becomes unblocked in the subsequent time step. By Lemma 2, the total swaps $H_{W}\left(t\right)$ could have made before time $T_{stable}$ is thus most $t^{*}+\left(n-m-j\right)\leq n-m$. Whenever an empty location is one step clockwise from $H_{W}\left(t\right)$, they will swap with probability at least 0.5 (the swap is not guaranteed, since it is possible the location is also adjacent to the head of another segment, and hence a tiebreaker will occur in regards to which segment head occupies the empty location in the next time step). Consequently, the expected number of time steps $H_{W}\left(t\right)$ is not adjacent to the head of another segment is bounded by 2(n−m). □

The proof of Theorem 1 now follows.

**Proof.**  Lemma 2 tells us that before time $T_{stable}$, |SW(t)| does not change in at most 2(n−m) time steps in expectation, whereas Lemma 1 tells us that the expected number of changes to |SW(t)| before time $T_{stable}$ is at most $m^{2}$. Hence, for any configuration of *m* locusts on a ring of track length *n*, $E\left[T_{stable}\right]\leq m^{2}+2\left(n-m\right)$.

Let us now show a locust configuration for which $E\left[T_{stable}\right]=\Omega \left(m^{2}+n\right)$, so as to asymptotically match the upper bound we found. Consider a ring with k=1, *m* divisible by 2, and an initial locust configuration where locusts are found at coordinates (0,1),(1,1),…(m/2,1) with a clockwise heading and at (−1,1),(−2,1),…(−m/2−1,1) with a counterclockwise heading, and the rest of the ring is empty. This is a ring with exactly two segments, each of size m/2. Since after every conflict, the segment sizes are offset by one in either direction, the expected number of conflicts between the heads of the segments that is necessary for stabilization is equal to the expected number of steps a random walk with absorbing boundaries at m/2 and −m/2 takes to end, which is $m^{2}/4$ (see [39]). Since the heads of the segments start at distance n−m from each other, it takes Ω(n−m) steps for them to reach each other. Hence the expected time for this ring to stabilize is $\Omega (m^{2}+n-m)$. □

### 4.2. Locusts on Wide Ringlike Arenas (k>1)

Let us now investigate the case where *m* locusts are marching on k>1 tracks of length *n*. The first question we should ask is whether, just as in the case of the k=1 setting, there exists some time *T* where all locusts have identical heading. The answer is “not necessarily”: consider for example the case k=2 where on the k=1 track, all locusts march clockwise, and on the k=2 track, all locusts march counterclockwise. According to the track-switching conditions (Section 3), no locust will ever switch tracks in this configuration; hence the locusts will perpetually have opposing headings. As we shall prove in this section, swarms stabilize *locally*–meaning that eventually, all locusts *on the same track* have identical heading, but this heading may be different between tracks.

Let us say that the *y*th track is stable if all locusts whose location is (·,y) have the identical heading. Note that once a track becomes stable, it remains this way forever, as by the model, the only locusts that may move into the track must have the same heading as its locusts. Let $T_{stable}$ be the first time when all the *k* tracks are stable. Our goal will be to prove the following asymptotic bounds on $T_{stable}$:

**Theorem** **2.** *$E\left[T_{stable}\right]=O\left(\min\left(\log\left(k\right)n^{2},mn\right)\right)$.*

Recalling Definition 1, each locust in the system belongs to some segment. Each track has its own segments. Locusts leave and join segments due to conflicts or when they pass from their current segment to a track on a different segment. In this section, we will treat segments as having persistent identities similar to SW in the previous section. We introduce the following notation:

**Definition** **5.** *Let S be a segment whose tail is A at some time $t_{0}$. We define S(t) to be the segment whose tail is A at the beginning of time t. If A is not a segment tail at time t, then we will say S(t)=∅ (this can happen once A changes its heading or moves to another track, or due to another segment merging with S(t) which might cause $b(A^{\leftarrow })$ to equal b(A), thus making A no longer the tail).*

*Furthermore, define $S_{1}$ to be the segment tail of S and $S_{i+1}=S_{i}^{\to }$.*

Let us give a few examples of the notation in Definition 5. Suppose at time $t_{1}$ we have some segment *S*. Then the tail of *S* is $S_{1}$, and the head is $S_{\left|S\right|}$. S(t) is the segment whose tail is $S_{1}$ at time *t*; hence $S(t_{1})=S$. Finally, $S{\left(t\right)}_{\left|S(t)\right|}$ is the head of the segment S(t).

In the k>1 setting, locusts can frequently move between tracks, which complicates our study of $T_{stable}$. Crucially, however, the number of segments on any individual track is non-increasing. This is because, first, as shown in the previous section, locusts moving and conflicting on the same track can never create new segments. Second, by the locust model, locusts can only move into another track when this places them between two locusts that already belong to some (clockwise or counterclockwise) segment.

That being said, locusts moving in and out of a given track make the technique we used in the previous section unfeasible. In the following definitions of *compact* and *deadlocked* locust sets, our goal is to identify configurations of locusts on a given track which locusts cannot enter from another track. Such configurations can be studied locally, focusing only on the track they are in. In the next several lemmas, we will bound the amount of time that can pass without either the number of segments decreasing or all segments entering into deadlock.

**Definition** **6.** *We call a sequence of locusts $X_{1},X_{2},\ldots$* ***compact*** *if $X_{i+1}=X_{i}^{\to }$ and either:*

*1.*  *every locust in X has a clockwise heading and for every i<|X|, $dist^{c}\left(X_{i},X_{i+1}\right)\leq 2$, or*
*2.*  *every locust in X has a counterclockwise heading and for every i<|X|, $dist^{cc}\left(X_{i},X_{i+1}\right)\leq 2$.*

*An unordered set of locusts is called compact if there exists an ordering of all its locusts that forms a compact sequence.*

**Figure 4:**
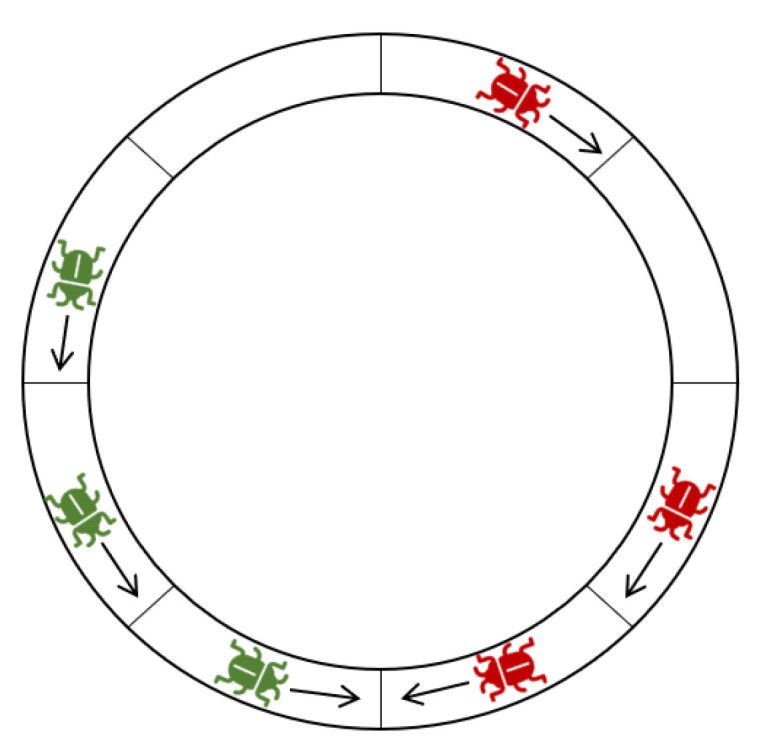
Two segments in deadlock, colored green and red (Definition 7).

**Definition** **7.** *Let $X=\{X_{1},X_{2},\ldots X_{j}\}$ and $Y=\{Y_{1},Y_{2},\ldots Y_{k}\}$ be two compact sets, such that the locusts of X have a clockwise heading and the locusts of Y have a counterclockwise heading. X and Y are* ***in deadlock** if $dist^{c}\left(X_{j},Y_{k}\right)=1$. (See Figure 4).*

A compact set of locusts *X* is essentially a platoon of locusts all on the same track which are heading in one direction and are all jammed together with at most one empty space between each consecutive pair. As long as *X* remains compact, no new locusts can enter the track between any two locusts of *X* because the model states that locusts do not move vertically into empty locations to which a locust is attempting to move horizontally, and the locusts in a compact set are always attempting to move horizontally to the empty location in front of them.

**Definition** **8.** *A maximal compact set is a set X such that for any locust A∉X, X∪A is not compact.*

A straightforward observation is that locusts can only belong to one maximal compact set:

**Observation** **1.** *Let A be a locust. If X and Y are maximal compact sets containing A, then X=Y.*

**Lemma** **5.** *Let X and Y be two sets of locusts in deadlock at the beginning of time t. Then at every subsequent time step, the locusts in X∪Y can be separated into sets $X^{\prime }$ and $Y^{\prime }$ that are in deadlock, or the locusts in X∪Y all have identical heading.*

**Proof.**  Let $X=\{X_{1},X_{2},\ldots X_{j}\}$ and $Y=\{Y_{1},Y_{2},\ldots Y_{k}\}$ be compact sets such that $X_{i+1}=X_{i}^{\to }$, $Y_{i+1}=Y_{i}^{\to }$. It suffices to show that if *X* and *Y* are in deadlock at time *t*, they will remain that way at time t+1, unless X∪Y’s locusts all have identical heading. Let us assume without loss of generality (“w.l.o.g.”) that *X* has a clockwise heading, and therefore *Y* has a counterclockwise heading. By the definition of deadlock, at time *t*, $X_{j}$ and $Y_{k}$ conflict, and the locust that loses joins the other set. Suppose w.l.o.g. that $X_{j}$ is the locust that lost. If |X|=1, then the locusts all have an identical heading, and we are finished. Otherwise, set $X^{\prime }=\left\{X_{1},\ldots X_{j-1}\right\}$ and $Y^{\prime }=\left\{Y_{1},Y_{2},\ldots Y_{k},X_{j}\right\}$. Note that since *X* and *Y* are compact at time *t*, no locust could have moved vertically into the empty spaces between pairs of locusts in X∪Y. Furthermore the locusts of *X* and *Y* all march toward $X_{j}$ and $Y_{k}$, respectively; hence the distance between any consecutive pair $X_{i},X_{i+1}$ or $Y_{i},Y_{i+1}$ could not have increased. Thus $X^{\prime }$ and $Y^{\prime }$ are compact.

To show that $X^{\prime }$ and $Y^{\prime }$ are deadlocked at time t+1, we just need to show that $dist^{c}\left(X_{j-1},X_{j}\right)$ is 1 at time t+1. Since the distances do not increase, if $dist^{c}\left(X_{j-1},X_{j}\right)$ was 1 at time *t*, we are finished. Otherwise $dist^{c}\left(X_{j-1},X_{j}\right)=2$ at time *t*, and since $X_{j}$ did not move (it was in a conflict with $Y_{k}$), $X_{j-1}$ decreased the distance in the last time step, hence it is now 1. □

**Lemma** **6.** *Suppose P and Q are the only segments on track K at time $t_{0}$, and P’s locusts have a clockwise heading. Let $d=dist^{c}\left(P_{1},Q_{1}\right)$. After at most 3d time steps, $P(t_{0}+3d)$ and $Q(t_{0}+3d)$ are in deadlock, or the track is stable.*

**Proof.**  The track K consists of locations of the form (x,y) for some fixed *y* and 1≤x≤n. For brevity, in this proof we will denote the location (x,y) simply by its horizontal coordinate, i.e., *x*, by writing (x)=(x,y).

We may assume w.l.o.g. that $t_{0}=0$ and that $P_{1}$ is initially at (0). Note that this means $Q_{1}$ is at (d) at time 0. If at any time t≤3d the track is stable, then we are finished, so we assume for contradiction that this is not the case. This means that $P_{1}$ and $Q_{1}$ do not change their headings before time 3d. This being the case, we get that $dist^{c}\left(P_{1},Q_{1}\right)$ is non-increasing before time 3d. Since the segments P(t) and Q(t) move toward each other at every time step t≤3d, we may focus only on the interval of locations [0,d], i.e., the locations (0),(1),…(d). We then define the distance dist(·,·) between two locusts in this interval whose *x*-coordinates are $x_{1}$ and $x_{2}$ as $|x_{1}-x_{2}|$.

**Figure 5:**
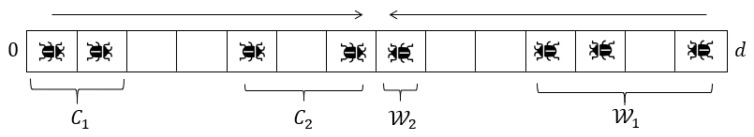
A partition into maximal compact subsets as in our construction. In this configuration, $L_{1}\left(t\right)=3$, $L_{2}\left(t\right)=3$, $L_{3}\left(t\right)=1$, and L(t)=7. Note that although $C_{1},C_{2}$ are compact, $P\left(t\right)=C_{1}\cup C_{2}$ is not compact, and similarly Q(t) is not compact; thus P(t) and Q(t) are not in deadlock, and L(t)≠1.

At any time t≤3d, we may partition the locusts in [0,d] into maximal compact sets of locusts. This partition is unique, by Observation 1. Let us label the maximal compact sets of locusts that belong to P(t) as $C_{1}^{t},C_{2}^{t},\ldots C_{c_{t}}^{t}$, where the segments are indexed from 1 to $c_{t}$, sorted by increasing *x* coordinates, such that $C_{1}^{t}$ contains the locusts closest to (0). Analogously, we label the maximal compact sets that belong to Q(t) as $W_{1}^{t},W_{2}^{t},\ldots W_{w_{t}}^{t}$, with indices running from 1 to $w_{t}$, sorted by decreasing *x*-coordinates such that $W_{1}^{t}$ contains the locusts that are closest to (d) (see Figure 5). In this proof, the distance between two sets of locusts X,Y, denoted dist(X,Y), is defined simply as the minimal distance between two locusts A∈X,B∈Y. Our proof will utilize the functions:

(1) $$\begin{array}{cc} L_{1}\left(t\right)=\sum_{i=1}^{c_{t}-1}dist\left(C_{i}^{t},C_{i+1}^{t}\right), & \;L_{2}\left(t\right)=\sum_{i=1}^{w_{t}-1}dist\left(W_{i}^{t},W_{i+1}^{t}\right)\\ L_{3}\left(t\right)=dist\left(C_{c_{t}}^{t},W_{w_{t}}^{t}\right), & \;L\left(t\right)=L_{1}\left(t\right)+L_{2}\left(t\right)+L_{3}\left(t\right) \end{array}$$

$L_{1}\left(t\right)$ is the sum of distances between consecutive clockwise-facing sets in the partition at time *t*. $L_{2}\left(t\right)$ is the sum of distances between the counterclockwise sets. $L_{3}\left(t\right)$ is the distance between the two closest clockwise and counterclockwise facing sets. The function L(t) is the sum of distances between consecutive compact sets in the partition. When L(t)=1, there are necessarily only one clockwise and one counterclockwise facing sets in the partition, which must equal P(t) and Q(t), respectively. Furthermore, L(t)=1 implies that the distance between P(t) and Q(t) is 1. Hence, when L(t)=1, P(t) and Q(t) are both in deadlock. The converse is true as well; hence L(t)=1 if and only if P(t),Q(t) are in deadlock. We will use L(t) as a potential or “Lyapunov” function [40] and show it must decrease to 1 within 3d time steps. By Lemma 5, once *P* and *Q* are in deadlock they will remain in deadlock until one of them is eliminated, which completes the proof.

Let us denote by max(X) the locust with maximum *x*-coordinate in *X*, and by min(X) the locust with minimal *x*-coordinate. We may also use max(X) and min(X) to denote the *x* coordinate of said locust. Note that $dist(C_{i}^{t},C_{i+1}^{t})$ is the distance between $max(C_{i}^{t})$ and $min(C_{i+1}^{t})$.

Recall that in the locust model, every time step is divided into a phase where locusts move horizontally (on their respective tracks), and a phase where they move vertically. First, let us show that the sum of distances $L_{1}\left(t\right)$ does not increase due to changes in either the horizontal or vertical phase. Since $L_{1}\left(t\right)$ is the sum of distances between compact partition sets whose locusts move clockwise, and for all $C_{i}^{t}$ except perhaps $C_{c_{t}}^{t}$, $max(C_{i}^{t})$ always moves clockwise, the distance $dist(C_{i}^{t},C_{i+1}^{t})$ does not increase as a result of locust movements (note that clockwise movements of $max(C_{i}^{t})$ do not result in a new compact set because the rest of the locusts in $C_{i}^{t}$ follow it). Furthermore, since conflicts cannot result in a new maximal compact set in the partition, conflicts do not increase $L_{1}\left(t\right)$. Hence, $L_{1}\left(t\right)$ does not increase in the horizontal phase. In the vertical phase, clockwise-heading locusts entering the track either create a new set in the partition, which does not affect the sum of distances (as they then merely form a “mid-point” between two other maximal compact sets), or they join an existing compact set, which can never increase $L_{1}\left(t\right)$. By the locust model, the only locusts that can move tracks are $max(C_{c_{t}}^{t})$ and $min(W_{w_{t}}^{t})$, since these are the only locusts for which the condition $b\left(A\right)\neq b\left(A^{\to }\right)$ is true, so locusts moving tracks cannot increase $L_{1}\left(t\right)$ either. In conclusion, $L_{1}\left(t\right)$ is non-increasing at any time step. By analogy, $L_{2}\left(t\right)$ is non-increasing.

Similar to $L_{1}$ and $L_{2}$, the distance $L_{3}\left(t\right)$ cannot increase as a result of locusts entering the track. It can increase as a result of a locust conflict which eliminates either $W_{w_{t}}^{t}$ or $C_{c_{t}}^{t}$, but such an increase is compensated for by a comparable decrease in either $L_{1}\left(t\right)$ or $L_{2}\left(t\right)$. It is also simple to check that, since P(t) and Q(t) are always moving toward each other when they are not in deadlock (i.e., when L(t)>1), there will be at least two compact sets in the partition that decrease their distance to each other; hence $L_{1},L_{2}$ or $L_{3}$ must decrease by at least one in the horizontal phase.

To conclude: $L_{1}\left(t\right)$ and $L_{2}\left(t\right)$ are non-increasing. $L_{3}\left(t\right)$ is non-increasing during the horizontal phase and as a result of new locusts entering K. If L(t)>1, L(t) decreases during each horizontal phase. Hence, L(t) decreases in every time step where L(t)>1, and no locusts in K move to another track.

What happens when locusts in K do move to another track? As proven, $L_{1}\left(t\right)$ and $L_{2}\left(t\right)$ do not increase. However, the distance $L_{3}\left(t\right)$ will increase, since the only locusts that can move tracks are $max(C_{w_{t}}^{t})$ and $min(W_{c_{t}}^{t})$. It is straightforward to check that when $C_{c_{t}}^{t}$ contains more than one locust, $L_{3}\left(t\right)$ will increase by at most two as a result of $max(C_{w_{t}}^{t})$ moving tracks. When $C_{c_{t}}^{t}$ contains exactly one locust, $L_{3}\left(t\right)$ can increase significantly (as $L_{3}\left(t\right)$ then becomes the distance between $C_{c_{t}-1}^{t}$ and $W_{w_{t}}$), but any increase is matched by the decrease in $L_{1}\left(t\right)$ as a result of $C_{c_{t}}^{t}$ being eliminated. Analogous statement hold for $W_{w_{t}}^{t}$, and hence $L_{3}\left(t\right)$ can increase by at most two as a result of one locust moving out of the track. We need to bound, then, the number of locusts in K that move tracks before time 3d. We define the potential function F(t):

(2) $$\begin{array}{c} F\left(t\right)=\sum_{i=1}^{c_{t}-1}\left(dist\left(C_{i}^{t},C_{i+1}^{t}\right)-1\right)+\sum_{i=1}^{w_{t}-1}\left(dist\left(W_{i}^{t},W_{i+1}^{t}\right)-1\right)+\left|P\left(t\right)\cup Q\left(t\right)\right|\\ =L_{1}\left(t\right)+L_{2}\left(t\right)-c_{t}-w_{t}+\left|P\left(t\right)\cup Q\left(t\right)\right| \end{array}$$

F(t) is the sum of the empty locations between consecutive compact sets in the partition whose locusts have the same heading plus the number of locusts in K. Note that F(t)≥0 at all times *t*. We will show F(t) is non-increasing and that it decreases whenever a locust leaves the track. Hence, at most F(0) locusts can leave the track.

Let us show that F(t) is non-increasing. We already know $L_{1}$ and $L_{2}$ are non-increasing. In the horizontal phase, |P(t)∪Q(t)| is of course unaffected. Then $c_{t}$ and $w_{t}$ can decrease as a result of maximal compact sets merging, hence increasing *F*, but this can only happen when the distance between two such sets has decreased; hence the resulting increase to *F* is undone by a decrease in $L_{1}$ and $L_{2}$. Hence, F(t) does not increase because of locusts’ actions during the horizontal phase.

Likewise, locusts leaving K can decrease $c_{t}$ or $w_{t}$ when they cause a maximal compact set to be eliminated, but this is matched by a comparable decrease in $L_{1}$ or $L_{2}$ which means that *F* does not increase due to locusts moving out of the track. Furthermore, |P(t)∪Q(t)| decreases when this happens. Hence, a locust moving out of the track decreases F(t) by at least one. Finally, let us show that locusts entering the track does not increase F(t).

At time *t*, locusts can only enter the track at empty locations that are found in intervals of the form $\left[max\left(W_{i}^{t}\right),min\left(W_{i+1}^{t}\right)\right]$ or $\left[max\left(C_{i+1}^{t}\right),min\left(C_{i}^{t}\right)\right]$ for some *i*. In particular, locusts cannot enter empty locations that are between two locusts belonging to the same compact set (because a locust in that set will always be attempting to move to that location in the next time step, and the model disallows vertical movements to such locations), nor can they enter the track on the empty locations between $min(C_{c_{t}}^{t})$ and $max(W_{w_{t}}^{t})$. Thus, locusts entering the track at time t decrease the amount of empty locations between two clockwise or counterclockwise compact partition sets (and perhaps cause the sets between which they enter to merge into a single compact set). This will always decrease $L_{1}\left(t\right)+L_{2}\left(t\right)-c_{t}-w_{t}$ by at least one and increase |P(t)∪Q(t)| by 1. On net, we see that new locusts entering K either decrease or do not affect *F*.

In conclusion, F(t) is non-increasing, and any time a locust moves to another track, F(t) decreases by one. Thus, at most F(0) locusts can move from K to another track. Recall that locusts moving out of the track can increase L(t) by at most two. Hence after at most L(0)+2F(0)≤d+2d=3d time steps, L(t)=1. □

**Lemma** **7.** *Let seg(t) denote the set of segments in all tracks at time t. At time t+3n, either every segment is in deadlock with some other segment, or |seg(t+3n)|<|seg(t)|.*

**Proof.**  Consider some track K and a segment *P* which is in that track at time *t*. Let us assume that |seg(t+3n)|=|seg(t)|, and show that P(t+3n) must be in deadlock with another segment. At any time $t^{\prime }\geq t$, as long as the number of segments on K does not decrease, the locusts of $P(t^{\prime })$ will be marching toward locusts of another segment, which we will label $Q(t^{\prime })$. They cannot collide or conflict with locusts belonging to any segment other than $Q(t^{\prime })$. Hence, other segments in K do not affect the evolution of P(t) and Q(t) before time t+3n, and we can assume w.l.o.g. that P(t) and Q(t) are the only segments in K at time *t*. Let *d* be as in the statement of Lemma 6. Since n≥d, Lemma 6 tells us that at some time $t\leq t^{*}\leq t+3n$, $P(t^{*})$ and $Q(t^{*})$ must be in deadlock. Since by Lemma 5, *P* and *Q* must remain in deadlock until one of them is eliminated, we see that at time t+3n they must still be in deadlock, since we assumed |seg(t)|=|seg(t+3n)|. □

**Theorem** **3.** *$E\left[T_{stable}\right]=O\left(mn\right)$.*

**Proof.**  Let |seg(t)| denote the number of segments at time *t*. $E[T_{stable}]$ can be computed as the sum of times $E[T_{2}+T_{4}+\ldots +T_{\left|seg(0)\right|}]$, where $T_{i}$ is the expected time until the number of segments drops below *i*, if it is currently *i* (we increment the index by two since segments are necessarily eliminated in pairs).

Let us estimate $E[T_{2i}]$. Suppose that at time *t*, the number of segments is 2i. Then after 3n steps at most, either the number of segments has decreased, or all segments are in deadlock. There are in total *i* pairs of segments in deadlock, and as there are *m* locusts, there must be a pair *P*, *Q* that contains at most min(m/i,n) locusts at time t+3n. By Lemma 5, *P*, *Q* remain in deadlock until either *P* or *Q* is eliminated. We can compute how long this takes in expectation, since at every time step after time t+3n, the heads of *P* and *Q* conflict, resulting in one of the segments increasing in size and the other decreasing. Hence, the expected time it takes *P* or *Q* to be eliminated is precisely the expected time it takes a symmetric random walk starting at 0 to reach either |P| or −|Q|, which is $\left|P|\cdot |Q|\leq \min({\left(\frac{m}{2i}\right)}^{2},{\left(\frac{n}{2}\right)}^{2}\right)$. Hence, $E\left[T_{2i}\right]\leq 3n+\min\left({\left(\frac{m}{2i}\right)}^{2},{\left(\frac{n}{2}\right)}^{2}\right)$.

Let us first assume m≥n. Using the fact that $\min\left({\left(\frac{m}{2i}\right)}^{2},{\left(\frac{n}{2}\right)}^{2}\right)={\left(\frac{n}{2}\right)}^{2}$ for i≤⌊m/n⌋, we have:

(3) $$\begin{array}{cc} E[T_{2}+T_{4}+\ldots +T_{\left|seg(0)\right|}] & \leq 3n\cdot \frac{\left|seg(0)\right|}{2}+\left\lfloor m/n\right\rfloor {\left(\frac{n}{2}\right)}^{2}+\sum_{i=\lceil m/n\rceil }^{\infty }{\left(\frac{m}{2i}\right)}^{2}\\ \; & \leq \frac{3}{2}mn+\frac{1}{4}mn+\frac{1}{4}m^{2}\sum_{i=0}^{\infty }{\left(\frac{1}{m/n+i}\right)}^{2}\\ \; & \leq \frac{7}{4}mn+\frac{1}{4}m^{2}\left(\frac{n^{2}}{m^{2}}+\frac{n}{m}\right)\leq \frac{9}{4}mn \end{array}$$

where we used the inequalities $\sum_{i=0}^{\infty }{\left(\frac{1}{m/n+i}\right)}^{2}\leq \frac{n^{2}}{m^{2}}+\int_{i=0}^{\infty }{\left(\frac{1}{m/n+i}\right)}^{2}=\frac{n^{2}}{m^{2}}+\frac{n}{m}$ and |seg(0)|≤m. If m<n, by using the identity $\sum_{i=1}^{\infty }{\left(\frac{1}{i}\right)}^{2}=\frac{\pi^{2}}{6}$ we obtain:

(4) $$E\left[T_{2}+T_{4}+\ldots +T_{\left|seg(0)\right|}\right]\leq 3n\cdot \frac{\left|seg(0)\right|}{2}+\sum_{i=1}^{\infty }{\left(\frac{m}{2i}\right)}^{2}\leq \frac{3}{2}mn+\frac{\pi^{2}}{24}m^{2}\leq \left(\frac{3}{2}+\frac{\pi^{2}}{24}\right)mn$$

So we see that $E\left[T_{stable}\right]=O\left(mn\right)$. □

Next we wish to show that $E\left[T_{stable}\right]=O\left(\log\left(k\right)n^{2}\right)$. For this, we require the following result:

**Lemma** **8.** *Consider k independent random walks with absorbing barriers at 0 and 2n, i.e., random walks that end once they reach 0 or 2n. The expected time until* ***all** k walks end is $O(n^{2}\log\left(k\right))$.*

**Proof.**  First, let us set k=1 and estimate the probability that the one walk has not ended by time *t*. Let *P* be the transition probability matrix of the random walk, and let v be the vector describing the initial probability distribution of the location of the random walker. Then $vP^{t}$ is the probability distribution of its location after *t* time steps [41]. The evolution of $vP^{t}$ is well-studied and relates to “the discrete heat equation” [42]. The probability that the walk has not ended at time *t* is the sum $\sum_{i=1}^{2n-1}v\left(i\right)$. Asymptotically, this sum is bounded by $O(\lambda^{t})$, where $\lambda =cos(\frac{\pi }{2n})$ is the second largest eigenvalue of *P* (cf. [42]).

Returning to general *k*, let $T_{k}$ be a random variable denoting the time when all *k* walks end. By looking at the series expansion of cos(1/x), we may verify that for n>1, $cos\left(\frac{\pi }{2n}\right)<1-\frac{1}{n^{2}}$. From the previous paragraph, and because the walks are independent, we therefore see that

(5) $$Pr\left(T_{k}\geq t\right)=1-Pr{\left(T_{1}<t\right)}^{k}=1-{\left(1-O(\lambda^{t})\right)}^{k}=1-{\left(1-O({\left(1-\frac{1}{n^{2}}\right)}^{t})\right)}^{k}$$

Consequently, for $t\gg n^{2}$, the following asymptotics hold for some constant *C*:

(6) $$Pr\left(T_{k}\geq t\right)<1-{\left(1-Ce^{-t/n^{2}}\right)}^{k}$$

where we used the fact that ${\left(1+x/n\right)}^{n}\to e^{x}$ as n→∞. Note that $Pr\left(T_{k}\geq t+n^{2}\log\left(C\right)\right)<1-{\left(1-e^{-t/n^{2}}\right)}^{k}$. Hence:

(7) E[Tk]=∫0∞Pr(Tk>t)dt≤n2log(C)+∫0∞1−(1−e−t/n2)kdt=n2log(C)+∫0∞1−∑j=0kkj(−1)je−tj/n2dt=n2log(C)+−∑j=1kkj(−1)j∫0∞e−tj/n2dt=n2log(C)+−n2∑j=1kkj(−1)jj=O(n2log(k))

where we used the equality ∑j=1kkj(−1)jj=−∑j=1k1j≈log(k). □

**Theorem** **4.** *$E\left[T_{stable}\right]=O\left(\log\left(k\right)\cdot n^{2}\right)$.*

**Proof.**  Let $seg_{i}\left(t\right)$ denote the number of segments in track *i* at time *t*, and define $M_{t}=\max_{1\leq i\leq k}seg_{i}\left(t\right)$. Let us bound the expected time it takes for $M_{t}$ to decrease. Define the set K(t) to be all tracks that have $|M_{t}|$ segments at time *t*. Then $M_{t}$ decreases at the first time $t^{\prime }>t$ when all tracks in K(t) have had their number of segments decrease. We may bound this with the following argument: slightly generalizing Lemma 7 to hold for subsets of tracks (Lemma 7 holds not just for the set seg(t) but for the segments in a given subset of tracks, with the proof being virtually identical. Here we apply the Lemma to the subset K(t+3n).), if $M_{t}$ does not decrease after 3n time steps (i.e., $M_{t}=M_{t+3n}$), all tracks in K(t+3n) now have all their segments in deadlock. The number of deadlocked segment pairs at every track in K(t+3n) is $M_{t}/2$, so in every such track there is such a pair with at most $2n/M_{t}$ locusts. By Lemma 8, using a similar argument as Theorem 3, these pairs of deadlocked segments resolve into a single segment after at most $c\cdot \log\left(k\right){\left(\frac{2n}{M_{t}}\right)}^{2}$ expected time for some constant *c*. Hence, the number of expected time steps for $M_{t}$ to decrease is bounded above by $3n+c\log\left(k\right){\left(\frac{2n}{M_{t}}\right)}^{2}$.

$T_{stable}$ is the first time when $M_{t}=0$. Let us assume *n* is even for simplicity (the computation will hold regardless, up to rounding). We have that $M_{0}\leq n$, and $M_{t}$ decreases in leaps of two or more (since segments can only be eliminated in pairs). Hence, $T_{stable}$ is bounded by the amount of time it takes $M_{t}$ to decrease at most n/2 times. By linearity of expectation, this time can be bounded by summing $3n+c\log\left(k\right){\left(\frac{2n}{M_{t}}\right)}^{2}$ over $M_{t}=n,n-2,n-4,\ldots 2$:

(8) $$\begin{array}{cc} E[T_{stable}]\leq & \frac{n}{2}\cdot 3n+c\log\left(k\right){\left(\frac{2n}{n}\right)}^{2}+c\log\left(k\right){\left(\frac{2n}{n-2}\right)}^{2}+\ldots +c\log\left(k\right){\left(\frac{2n}{2}\right)}^{2}\\ \leq & \frac{3}{2}n^{2}+4c\log\left(k\right)n^{2}\sum_{i=1}^{\infty }{\left(\frac{1}{2i}\right)}^{2}=\frac{3}{2}n^{2}+\frac{\pi^{2}}{6}c\log\left(k\right)n^{2}=O\left(\log\left(k\right)n^{2}\right) \end{array}$$

as claimed. □

The proof of Theorem 2 follows immediately from Theorems 3 and 4 by taking the minimum.

#### Erratic Track Switching and Global Consensus

Theorem 2 shows that, after finite expected time, all locusts on a track have an identical heading. This is a stable *local* consensus, in the sense that two different tracks may have locusts marching in opposite directions forever. We might ask what modifications to the model would force a *global* consensus, i.e., make it so that stabilization occurs only when all locusts across *all* tracks have the identical heading. There is in fact a simple change that would force this to occur. Let us assume that at time step *t* any locust has some probability of acting “erratically” in either the vertical or horizontal phases:

1. With probability *r*, a locust might behave erratically in the horizontal phase, staying in place instead of attempting to move according to its heading.
2. With probability *p*, a locust may behave erratically in the vertical phase, meaning that even if the vertical movement conditions (1)–(3) of the model (see Section 3) are not fulfilled, the locust attempts to move vertically to an adjacent empty space on the track above or below them (if such empty space exists).

These behaviors are independent, and so a locust may behave erratically in both the vertical and horizontal phases, in just one of them, or in neither.

The next theorem shows that the existence of erratic behavior forces a global consensus of locust headings. The goal is to prove that there is some finite time after which all locusts must have the same heading. Note that the bound we find for this time is crude and is not intended to approximate $T_{stable}$. We study the question of how *p* affects $T_{stable}$ empirically in the next section.

**Theorem** **5.** *Assuming there is at least one empty space (i.e., m<nk), and the probability of erratic track switching is 0<r,p<1, the locusts all have identical heading in finite expected time.*

**Proof.**  Our goal is to show that all locusts must have identical heading in finite expected time. We will find a crude upper bound for this time. It suffices to show that as long as there are two locusts with different headings in the system (perhaps not on the same track), there is a bounded-above-zero probability *q* that within a some constant, finite number of time steps *C* (and shall show $C=O(\log\left(k\right)n^{2}+nk)$), the number of locusts with a clockwise heading will increase. This amounts to showing that there is a sequence of events, each individual event happening with non-zero probability, that culminates in a conflict between two locusts occurring (since any conflict has probability 0.5 of increasing the number of clockwise locusts). Since q>0, the only stable state of locust headings is the state where all locusts have the identical heading, as otherwise there is always some probability that all locusts will have a clockwise heading after m·C time steps. This completes the proof.

Let us show such a sequence of events. First let us consider the case where there is a track in which two locusts have non-identical headings. In this case, assuming no locusts behave erratically for $O(\log\left(k\right)n^{2})$ steps (which occurs with a tiny but bounded-above-zero probability since p,r>0), Theorem 2 tells us that in expected $O(\log\left(k\right)n^{2})$ steps, locusts on the same track will have the identical heading. Hence, there is a sequence of events that happens with non-zero probability which leads to local consensus in the tracks.

If any conflict occurs during this sequence, we are finished. Otherwise, we need to show a sequence of events that leads to a conflict, assuming all tracks are stable. The only thing that causes locusts in local consensus to move tracks is erratic behavior. If two adjacent tracks have locusts with non-identical heading, and there is at least one empty space in one of them, then (since r>0) with some probability within at most *n* time steps an empty space in one track will be vertically adjacent to a locust in the other track. At this point, with probability *p*, that locust will move from one track to the other. This creates a situation where in one track there are locusts of different headings again. If the erratic locust moves tracks at the right time, upon moving it will be adjacent to another locust in its new track, whose heading is different. Hence, the erratic locust will enter a conflict in the next time step, which will increase the number of clockwise locusts with probability 0.5.

Now let us consider a pair of two adjacent tracks with locusts of different headings such that there no empty space in one of them. We note that since there is at least one empty location in *some* track, erratic behavior can cause that empty location to move vertically in an arbitrary fashion until, after at most *k* movements, it enters a track from the pair. With non-zero probability, this can take at most nk time steps, after which we are reduced to the situation in the previous paragraph.

A pair of adjacent tracks that have locusts with different headings must exist unless there is global consensus. Hence, in every $O(\log\left(k\right)n^{2}+nk)$ time steps where there is no global consensus, there is a some probability q>0 that the number of clockwise-heading locusts will increase. □

## 5. Simulation and Empirical Evaluation

Let us explore some questions about the expected value of $T_{stable}$ through numerical simulations. Certain aspects of the locusts’ dynamics were not studied in our formal analysis, the most interesting of which is the helpful effects of track switching on $T_{stable}$. Recall that our model allows locusts to switch tracks if this would enable them to avoid a conflict and join a track where *locally*, locusts are marching in their same direction. At least in principle, this seems like it should help our locusts achieve local stability faster, hence decrease $T_{stable}$. However, recall also that we do not specify *when* locusts switch tracks, which means that some locusts might never switch tracks, or they might choose to do so in the worst possible moments. Hence, the positive effect track-switching usually has on $T_{stable}$ cannot be reflected in the bounds we found for $E[T_{stable}]$, since these bounds must reflect all possible locust behaviors. Under ordinary circumstances, however, it seems as though frequent track switching should noticeably decrease the time to local stabilization. As we shall see numerically, this is indeed the case. This justifies the track-switching behavior as a mechanism that, despite being highly local, enables the locusts to achieve local consensus about the direction of motion sooner.

**Figure 6:**
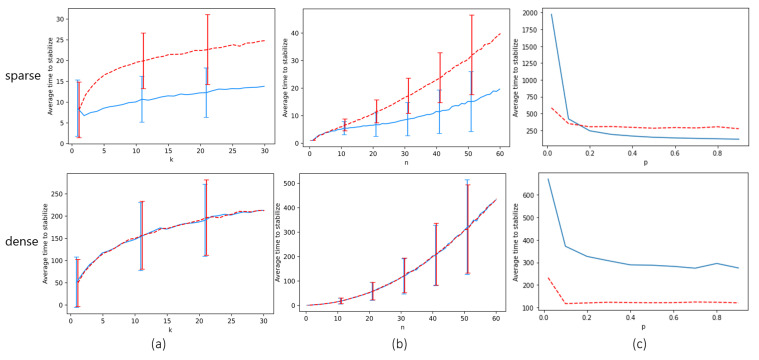
Simulations of the locust model. The *y* axis is $T_{stable}$. Column (**a**) measures $T_{stable}$ for k=1…30, with *n* fixed at 30. Column (**b**) measures $T_{stable}$ for n=1…60, with *k* fixed at 5. Column (**c**) measures $T_{stable}$ with n=30,k=5, and *p* (the probability of erratic behavior) going from 0 to 1. The top row measures $T_{stable}$ in sparse locust configurations (m≈0.1n), while the bottom row does so for dense configurations (m≈0.5n). The dashed red line estimates $T_{stable}$ when locusts never switch tracks (except while behaving erratically in column (**c**)); the blue line estimates $T_{stable}$ when locusts switch tracks as often as the model rules allow. Error bars show the standard deviations.

In Figure 6a,b, we measure $T_{stable}$ as it varies with *n* and *k*, assuming the probabilities of erratic behavior are 0 (i.e., r=p=0). We simulate two different locust configurations: a “dense” configuration and a “sparse” configuration. In the dense configuration, 50% of locations are initiated with a locust, with the locations chosen at random. In the sparse configuration, 10% of locations are initiated with a locust (or slightly more, to guarantee all tracks start with two locusts). The locusts are initiated with random heading. We measure the effect of track switching on $T_{stable}$; the opaque lines measure $T_{stable}$ when locusts switch tracks as often as they can (while still obeying the rules of the model), and the dotted lines measure $T_{stable}$ when locusts never switch tracks. For every value of *n*, *k*, we ran the simulation 2000 times and averaged $T_{stable}$ over all simulations.

As we can see, in the sparse configuration, track-switching has a significantly positive effect on time to stabilization. For example, with k=30, n=30, $T_{stable}$ is approximately 13.5 when locusts switch tracks as soon as they can and approximately 25 when they never switch tracks—nearly double. In the dense configuration, we see that enabling locusts to move tracks has little to no effect, since the locust model rarely allows them to do so due to the tracks being overcrowded.

In column (c) of Figure 6, we measure how a non-zero probability *p* of erratic behavior affects $T_{stable}$. We set r=0. As we proved in the previous section, whenever p>0, stabilization requires *global* rather than local consensus. Hence, we cannot directly compare the $T_{stable}$ of these graphs with columns (a) and (b), where $T_{stable}$ measures the time to local consensus. We note that the expectation and variance of $T_{stable}$ approach *∞* as *p* goes to 0, since when p=0, global stability can never occur in some initial configurations. $E[T_{stable}]$ decreases sharply as *p* goes to some critical point around 0.1, and decreases at a slower rate afterwards. It is interesting to note that low probability of erratic behavior affects $E[T_{stable}]$ significantly more in the *sparse* configuration, where for p=0.02, if locusts also switch tracks whenever the model allows them, $E[T_{stable}]$ was measured as being approximately 1974, as opposed to 669 in the dense configuration. One of the core reasons for this seems to be that in the sparse configuration, when a locust erratically moves to a track with a lot of locusts not sharing its heading, it will often be able to *non-erratically* move back to its former track, thus preventing locust interactions between tracks of different headings. When we disabled the locusts’ ability to switch tracks non-erratically, $T_{stable}$ was significantly smaller in the sparse configuration ($E[T_{stable}]\approx 232$ for p=0.02).

Based on the above, we make the curious observation that, while non-erratic track switching accelerates local consensus, for some track-switching behaviors it will in fact decelerate the attainment of global consensus. This is seen by the fact that frequent non-erratic track-switching was helpful in Columns (a) and (b) of Figure 6 but increased time to stabilization in Column (c). This is perhaps a very natural observation because agents that aggressively switch tracks will attempt to avoid conflict as often as possible, whereas conflict is necessary to create global consensus.

**Figure 7:**
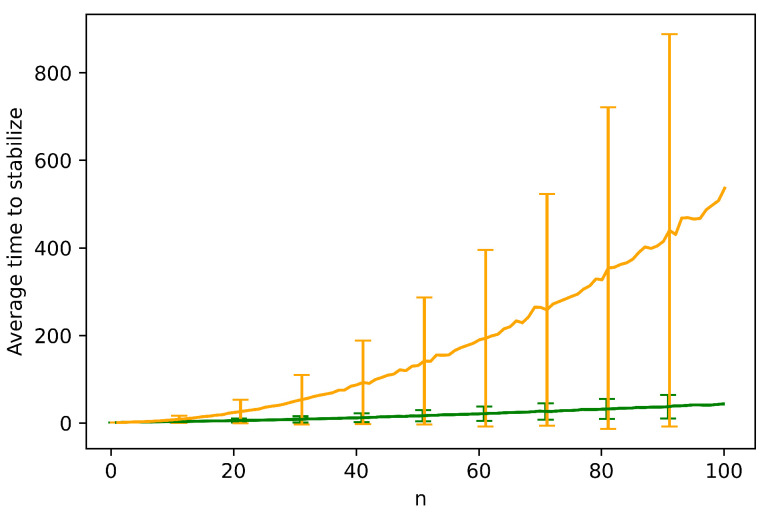
Simulations of the locust model fixing k=1 and letting *n* run from 1 to 100. The *y* axis is $T_{stable}$. The orange line denotes dense locust configurations (m≈0.5n), and the green line denotes sparse configurations (m≈0.1n). Error bars show the standard deviations.

To finish this section, we also verify the bounds of Theorem 1 by numerical simulation, by fixing k=1 and measuring $T_{stable}$ as *n* goes from 1 to 100—see Figure 7. We again measure both sparse and dense configurations (i.e., m≈0.1n and m≈0.5n, respectively). The average expected time appears asymptotically bounded by $m^{2}$, as expected. We also simulated the asymptotic worst-case locust configuration in the proof of Theorem 1 (not illustrated in Figure 7) and confirmed its stabilization time is asymptotically $\Omega (m^{2}+n-m)$, verifying that the bounds of Theorem 1 are asymptotically tight.

## 6. Concluding Remarks

We studied collective motion in a model of discrete locust-inspired swarms, and bounded the expected time to stabilization in terms of the number of agents *m*, the number of tracks *k*, and the length of the tracks *n*. We showed that when the swarm stabilizes, there must be a local consensus about the direction of motion. We also showed that, when the model is extended to allow a small probability of erratic behavior to perturb the system, global consensus eventually occurs.

A direct continuation of our work would be to find upper bounds on time to stabilization when there is some probability of erratic behavior. Furthermore, our empirical simulations suggest several curious phenomena related to erratic behavior. First, there seems to be a clash between “erratic” and non-erratic, “rational” track-switching, as when locusts switch tracks non-erratically to avoid collisions. This seems to accelerate the attainment of local consensus but mostly hinder the attainment of global consensus. Second, increasing the probability of erratic track-switching *p* behavior was helpful in accelerating global consensus up to a point, but in simulations, its impact seemed to fall off past a small critical value of *p*. In future work, it would be interesting to investigate these “phase transition” aspects of the model.

As discussed in the Related Work section, in [18,19] it is observed that at intermediate densities, swarms of locusts exhibit periodic directional switching, and at low densities the directions of motion are random. Although this phenomenon does not occur in our model, if we assume each locust has a small probability r>0 of randomly flipping their heading at the beginning of a time step, such directional switching becomes possible, with probability inversely proportional to the density (or so we expect). This extension of our model, of course, does not have stable states, thus cannot be studied by the same methods we used in this work. Nevertheless, we would be interested in studying it in terms of the *expected time* the swarm spends in consensus or near-consensus about the direction of motion before directional switching occurs.

For the sake of mathematical theory, the authors would be very interested in rigorous results established over a fully asynchronous version of this model where locust wake-up times are determined independently. In such a model, the winner of a conflict between two locusts can be determined as the locust that wakes up first (thus exerts pressure on the other locust first), which is perhaps more elegant. We speculate that most of the conclusions will not be majorly affected by transitioning to an asynchronous model.

Although our agent marching model is inspired by experiments on locusts, it can be understood in more abstract terms as a model that describes a situation where many agents that wish to maintain a direction of motion are confined to a small space where they exert pressure on each other. It is natural to ask what kinds of collective dynamics, if any, we should expect when this small space has a different topology; rather than a ringlike arena, we might consider, e.g., a square arena. We believe that rich models of swarm dynamics can be discovered through observing natural organisms exert pressure on each other in such environments. In the introduction, we mentioned points of similarity between our model and models of opinion dynamics. We suspect that these points of similarity will remain in settings with non-ringlike arenas and might provide a starting point for formally modeling and analyzing them.

## Data Availability

Not applicable.
